# Arctigenin derivative (ARC-18) improved mitochondrial dysfunction and ameliorated frataxin deficiency symptoms via PGC-1α signaling

**DOI:** 10.1016/j.gendis.2025.101838

**Published:** 2025-09-01

**Authors:** Qichao Gong, Xiao Han, Tiansu Liu, Bocheng Xiong, Bingge Zhang, Yongmei Xie, Huali Wan, Tahir Ali, Xifei Yang, Shupeng Li

**Affiliations:** a State Key Laboratory of Chemical Oncogenomics, School of Chemical Biology and Biotechnology, Peking University Shenzhen Graduate School, Shenzhen, Guangdong 518055, China; b Shenzhen Key Laboratory of Modern Toxicology, Shenzhen Medical Key Discipline of Health Toxicology (2020–2024), Shenzhen Center for Disease Control and Prevention, Shenzhen, Guangdong 518055, China; c Department of Toxicology, School of Public Health, Shanxi Medical University, Taiyuan, Shanxi 030001, China; d School of Public Health, Key Laboratory of Environmental Pollution Monitoring and Disease Control, Ministry of Education, Guizhou Medical University, Guiyang, Guizhou 561113, China; e State Key Laboratory of Biotherapy and Cancer Center, West China Hospital, Sichuan University, and Collaborative Innovation Center of Biotherapy, Chengdu, Sichuan 610041, China; f Department of Laboratory Medicine, Guangdong Provincial People's Hospital (Guangdong Academy of Medical Sciences), Southern Medical University, Guangzhou, Guangdong 510000, China; g Shenzhen Bay Laboratory, Shenzhen, Guangdong 518055, China

Friedreich's ataxia (FA), a progressive neurodegenerative disorder, stems from Frataxin (FXN) gene mutations, leading to a deficiency in frataxin, a crucial protein for mitochondrial function and iron–sulfur cluster biogenesis.^1^ Frataxin loss impacts key regions of the central nervous system (CNS), like the cerebellum and spinal cord, contributing to neurological deficits that can involve destabilization of the actin cytoskeleton and hinder axonal regeneration in affected neurons.^2^ These underlying mechanisms highlight potential therapeutic avenues, with mitochondrial restoration being a key focus, although the complex issue of tissue-specific iron dysregulation presents challenges for treatment development.^3^ Notably, PGC-1α, a key regulator of mitochondrial biogenesis, could be diminished in FA, making it a promising therapeutic target.^4^ However, arctigenin (ATG) activates PGC-1α; its poor solubility and extensive metabolism limit its clinical use.^5^ Herein, we developed ARC-18, a novel ATG derivative with enhanced pharmacological properties (Supplementary data). ARC-18 effectively restored mitochondrial function by restoring the levels of key proteins involved in mitochondrial dynamics (DRP1, OPA1, and MFN1) and biogenesis/mitophagy (PINK1/Parkin) and respiratory complex activity in cellular and mouse models of FA with frataxin deficiency. Concurrently, ARC-18 improved motor coordination and neuronal pathology in YG8R mice, which was dependent on the activation of PGC-1α. Mechanistically, FXN deficiency suppressed PGC-1α, while ARC-18 treatment restored its expression; this ARC-18 effect was abated by SR-18292 (a PGC-1α inhibitor), confirming pathway dependence.

Initially, we knocked down *FXN* in N2a cells (a neuronal model), which resulted in disrupted mitochondrial dynamics (↑DRP1, ↓MFN1/OPA1), elevated reactive oxygen species (ROS), reduced mtDNA copy numbers, and down-regulated PGC-1α and p-AMPKα in the FXN-deficient N2a cells (Fig. 1A and B; Fig. S1). This notion was aligned with our proteomic analysis of the cerebellums of mice (WT/YG8R) (Fig. 1F). However, ARC-18 treatment restored the expression of key mitochondrial proteins (PGC-1α, FXN, SDHB, COX5A, and UQCRFS1) (Fig. 1C and D), indicating functional recovery. Notably, co-treatment with the PGC-1α inhibitor SR-18292 abolished ARC-18 effects (Fig. S2B), confirming its dependence on PGC-1α activation to rescue mitochondrial deficits in FXN-deficient cells.

**Figure 1:**
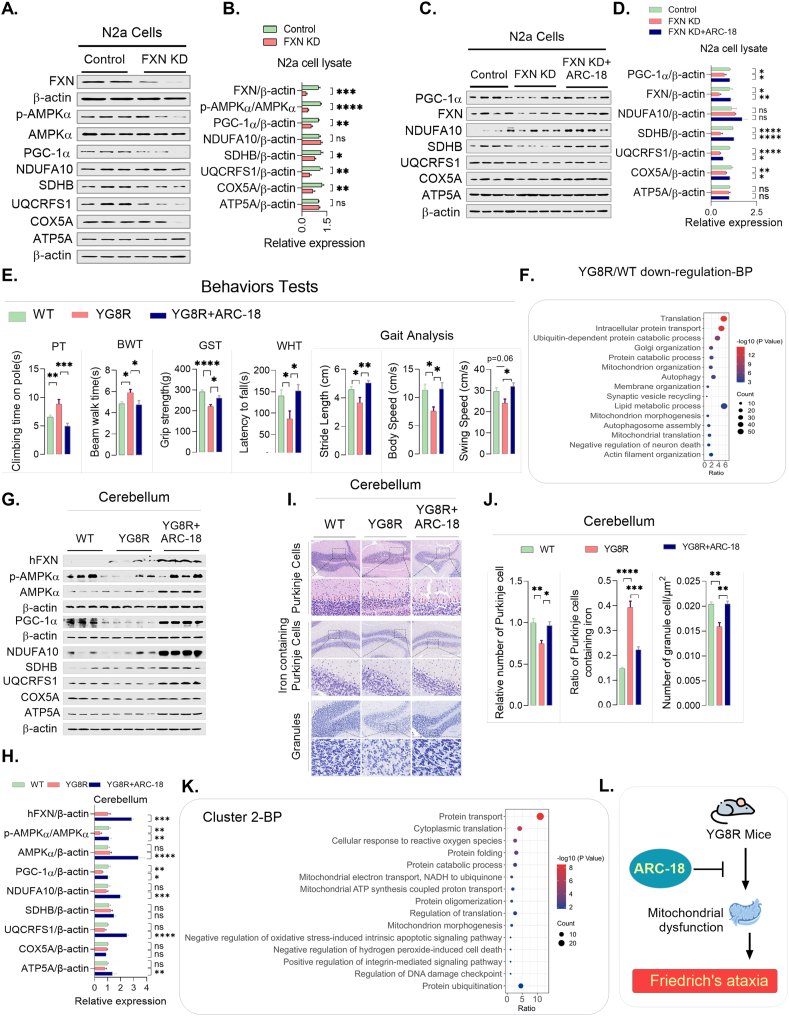
ARC-18 ameliorated mitochondrial dysfunction and behavioral deficits in the Friedrich's ataxia mouse model. *In vitro* validation in FXN-knockdown (KD) N2a cells: **(A**–**D)** Immunoblot analysis of cell lysates demonstrating ARC-18–mediated restoration of the following: Frataxin (FXN) expression, Mitochondrial protein expression (SDHB, UQCRFS1 and COX5A), and PGC-1α levels and AMPK signaling activation in the control, FXN-KD, and FXN-KD + ARC-18 groups. *n* = 4–9. *In vivo* validation in YG8R mice: **(E)** Behavioral assessments in the YG8R (FA model) and YG8R + ARC-18 cohorts, *n* = 8–19. Motor coordination: Pole climbing time (PT), beam walking test (BWT); Muscle strength: Grip strength test (GST), wire hanging test (WHT); Gait analysis: Stride length, body speed, swing speed. **(F)** Proteomic analysis of cerebellar tissue in WT and YG8R mice. *n* = 4. **(G, H)** Western blot of cerebellar lysates showing ARC-18-dependent recovery of mitochondrial proteins, AMPK/PGC-1α pathway activity and FXN levels in the WT, YG8R, and YG8R + ARC-18 groups, *n* = 4–8. **(I, J)** Histopathological and biochemical analyses revealed that cerebellar Purkinje cell loss and iron accumulation resulted in a reduction in Granule cell density in YG8R mice, which was rescued by ARC-18. *n* = 3–4. **(K)** Proteomic analysis confirmed the therapeutic effects of ARC-18, aligning with the *in vivo* results. *n* = 4. **(L)** Schematic model summarizing ARC-18's mechanism of action in FA. Data are presented as mean ± SEM. Two-group comparisons were performed via Student's *t*-test; Multi-group comparisons were performed via one-way ANOVA with Tukey's post hoc test. Significance: *P* < 0.05, ∗*P* < 0.05, ∗∗*P* < 0.01, ∗∗∗*P* < 0.001, ∗∗∗∗*P* < 0.0001; ns, not significant.

To evaluate the *in vivo* efficacy of ARC-18 in FA, we treated YG8R mice orally with ARC-18 (32.9 mg/kg) for 8 weeks. ARC-18 showed superior metabolic stability (prolonged metabolic half-life over arctigenin, as detailed in the Supplementary data) and effectively reversed FXN deficiency-induced mitochondrial dysfunction in the cerebellum. Specifically, ARC-18 treatment significantly increased the expression of genes encoding key regulators of mitochondrial dynamics (OPA1, DRP1), biogenesis (PGC-1α), electron transport chain (ETC) components (NDUFA10, UQCRFS1, and ATP5A), and mitophagy (PINK1, Parkin). Furthermore, ARC-18 treatment increased p-AMPKα, Beclin-1 and LC3B-II expressions (Fig. 1G and H; Fig. S6A), indicating that ARC-18 could restore mitochondrial function, possibly via autophagic flux. Moreover, PGC-1α was dysregulated in the cerebellum of YG8R mice, underscoring PGC-1α up-regulation as a promising neuroprotective strategy in FA. Strikingly, ARC-18 (acted as PGC-1α agonist) restored FXN levels and up-regulated PGC-1α, subsequently increasing mitochondrial protein expression (Fig. 1G and H; Fig. S6A). In addition to restoring mitochondrial function and improving motor performance, ARC-18 exhibited significant anti-inflammatory and antioxidant effects, targeting key pathological features of neurodegenerative diseases where inflammation, oxidative stress, and mitochondrial dysfunction are interconnected (Fig. S7A and S7B).

The YG8R mouse model, a well-established preclinical model of FA characterized by FXN deficiency, progressively develops significant neurodegeneration and pronounced motor impairments that mirror the human condition.^1^ Herein, YG8R mice exhibited marked motor incoordination, manifested as prolonged times in climbing and beam walk tasks, a demonstrable reduction in grip strength, and a significantly shorter latency to fall in wire-hanging assessments. Furthermore, YG8R mice displayed characteristic gait abnormalities, including decreased stride length, body speed, and swing time, and increased stand time and paw drag (Fig. 1E; Fig. S3A). Moreover, histological analysis revealed marked neurodegeneration and systemic pathology, including cerebellar Purkinje cell loss with iron accumulation, reduced granule cell density, spinal motor neuron degeneration, cardiac tissue disorganization, and pronounced skeletal muscle atrophy in YG8R mice (Fig. 1I and J; Fig. S3B).

Notably, an 8-week treatment regimen with ARC-18 substantially ameliorated of these debilitating deficits in YG8R mice. Compared with YG8R mice, ARC-18-administered mice exhibited a significant improvement in motor coordination, as quantitatively evidenced by a reduction in climbing time (↓44%, *P* = 0.0002), a faster traversal of the beam (↓19%, *P* = 0.022), a measurable enhancement in grip strength (↑18%, *P* = 0.034), and a prolonged endurance in the wire-hanging test (↑75%, *P* = 0.042). Comprehensive gait analysis further revealed that ARC-18 treatment facilitated recovery towards normal gait parameters, restoring stride length (↑38%, *P* = 0.007), body speed (↑51%, *P* = 0.005), and swing speed (↑33%, *P* = 0.034) to near-physiological levels, and concurrently reducing stand time (↓28%, *P* = 0.031) and the incidence of paw drag (↓33%, *P* = 0.039). Critically, ARC-18 treatment did not affect limb loading or the print area (Fig. 1E; Fig. S3A).

Furthermore, a detailed histological examination demonstrated that ARC-18 exerted significant neuroprotective effects within the cerebellum, leading to a notable increase in Purkinje cell density (*P* = 0.010) and a marked reduction in iron-laden Purkinje cells (*P* = 0.0007). Additionally, ARC-18 treatment positively impacted the cerebellar granular layer, as evidenced by an increase in the number of granule cells (*P* = 0.0016). In the spinal cord, ARC-18 treatment contributed to the preservation of motor neurons (*P* = 0.0023), and within the heart, it mitigated the pathological enlargement of cells (*P* = 0.010). Furthermore, analysis of skeletal muscle revealed that ARC-18 effectively attenuated muscle fiber atrophy and improved the gap width within the gastrocnemius muscle (*P* = 0.030) (Fig. 1I and J; Fig. S3B), suggesting the therapeutic efficacy of ARC-18 against peripheral neuropathy-associated muscle wasting.

In addition, our proteomic analysis revealed that ARC-18 ameliorates mitochondrial dysfunction in FXN-deficient models by modulating key metabolic and stress-response pathways. Treatment with ARC-18 significantly enhanced oxidative phosphorylation (OXPHOS), mitochondrial proteostasis, and structural integrity while suppressing glycolysis, apoptosis, and nitrosative stress. Notably, the up-regulation of PGC-1α-mediated pathways suggests a mechanistic basis for the restored mitochondrial function, consistent with ARC-18's therapeutic potential in FA. These findings align with our *in vivo* results, showing that ARC-18 restores mitochondrial electron transport, morphogenesis and proteostasis while suppressing glycolysis, apoptosis and oxidative stress in the YG8R mouse cerebellum (Fig. 1K; Figs. S4, S5, S6B).

In conclusion, this study provides compelling evidence that ARC-18 holds significant potential as a therapeutic candidate for FA. By targeting PGC-1α-mediated mitochondrial recovery and addressing multiple facets of FA pathogenesis, ARC-18 demonstrates a unique potential for disease modification (Fig. 1L; Fig. S8). Given its oral bioavailability and significant neuroprotective effects, ARC-18 warrants further clinical evaluation to assess its safety and efficacy in human subjects. The findings of this study underscore the therapeutic potential of PGC-1α-targeted therapies in treating mitochondrial diseases and advocate for the continued development of ARC-18 as a disease-modifying treatment for FA, potentially offering a new avenue for addressing this debilitating disorder.

## CRediT authorship contribution statement

**Qichao Gong:** Methodology, Investigation, Formal analysis, Conceptualization. **Xiao Han:** Methodology. **Tiansu Liu:** Methodology. **Bocheng Xiong:** Methodology. **Bingge Zhang:** Methodology. **Yongmei Xie:** Resources, Funding acquisition. **Huali Wan:** Writing – review & editing, Resources, Funding acquisition. **Tahir Ali:** Writing – review & editing, Writing – original draft, Methodology. **Xifei Yang:** Writing – review & editing, Supervision, Funding acquisition. **Shupeng Li:** Writing – review & editing, Visualization, Validation, Supervision, Conceptualization.

## Ethics declaration

All experimental procedures were carried out according to the protocols approved by the Institutional Animal Care and Use Committee of Peking University Shenzhen Graduate School and Shenzhen Center for Disease Control and Prevention, Shenzhen, Guangdong, China. (Approval Number: 11110).

## Data availability

All data generated or analyzed during this study are included in this published article and supplementary data.

## Funding

This work was supported by the Key Basic Research Program of Shenzhen Science and Technology Innovation Commission (China) (No. JCYJ20200109150717745), Science and Technology Department of Guizhou Province, China [Support (2021) general 117 and Achievements (2022) general 014], the NSFC Incubation Project of Guangdong Provincial People's Hospital (China) (No. KY0120220045), the Science and Technology Program of Guangzhou, China (No. 2023A04J0508) and Shenzhen Medical Research Fund (China) (No. D2403015).

## Conflict of interests

All the authors declare no conflict of interests.
