# Supplementary material for: Arctigenin derivative (ARC-18) improved mitochondrial dysfunction and ameliorated frataxin deficiency symptoms via PGC-1α signaling
Source: Genes Dis. 2025 Sep 1;13(4):101838. doi: 10.1016/j.gendis.2025.101838 (PMC13011025; doi:10.1016/j.gendis.2025.101838)
Supplement: Multimedia component 1 [file mmc1.docx]

**Supplementary Data 3. Detailed antibody information**

| Antibody | Company | Lot Number | Dilute | Source |
| --- | --- | --- | --- | --- |
| mFXN | Proteintech | 14147-1-AP | 1/1000 | Rabbit |
| Drp1 | Cell signaling technology | 8570 | 1/1000 | Rabbit |
| Mfn1 | Santa Cruz | sc-166644 | 1/500 | Mouse |
| OPA1 | Cell signaling technology | 80471 | 1/1000 | Rabbit |
| Beclin-1 | Cell signaling technology | 3738 | 1/1000 | Rabbit |
| LC3B | Abcam | ab48394 | 1/1000 | Rabbit |
| Nrf2 | Cell signaling technology | 12721 | 1/1000 | Rabbit |
| HO-1 | Cell signaling technology | 70081 | 1/1000 | Rabbit |
| NFS1 | Abcam | ab229829 | 1/1000 | Rabbit |
| p-AMPKα(Thr172) | Cell signaling technology | 2535 | 1/1000 | Rabbit |
| AMPKα | Cell signaling technology | 5832 | 1/1000 | Rabbit |
| PGC1α | Abcam | ab54481 | 1/1000 | Rabbit |
| NDUFA10 | Abcam | ab174829 | 1/1000 | Rabbit |
| SDHB | Abcam | ab14714 | 1/1000 | Mouse |
| UQCRFS1 | Abcam | ab14746 | 1/1000 | Mouse |
| COX5A | Abcam | ab110262 | 1/1000 | Mouse |
| ATP5A | Abcam | ab14748 | 1/1000 | Mouse |
| PINK1 | Abcam | ab23707 | 1/1000 | Rabbit |
| Parkin | Cell signaling technology | 4211S | 1/1000 | Mouse |
| ATG5 | Cell signaling technology | 12994 | 1/1000 | Rabbit |
| P62 | Cell signaling technology | 5114 | 1/1000 | Rabbit |
| NeuN | Sigma | MAB377 | 1/500 | Mouse |
| IBA1 | Cell signaling technology | 17198S | 1/1000 | Rabbit |
| GFAP | Cell signaling technology | 3670S | 1/1000 | Mouse |
| SOD2 | Cell signaling technology | 13194 | 1/1000 | Rabbit |
| GPX4 | Invitrogen | PA5-102521 | 1/1000 | Rabbit |
| Actin | Santa Cruz | sc-47778 | 1/500 | Mouse |
