# Supplementary material for: Arctigenin derivative (ARC-18) improved mitochondrial dysfunction and ameliorated frataxin deficiency symptoms via PGC-1α signaling
Source: Genes Dis. 2025 Sep 1;13(4):101838. doi: 10.1016/j.gendis.2025.101838 (PMC13011025; doi:10.1016/j.gendis.2025.101838)
Supplement: Multimedia component 2 [file mmc2.pdf]

Supplementary Tables

Supplementary Table 1. Drug concentration changes in the plasma after a single oral gavage of **ARC-18**(35 mg/kg, 0.058 mol) in Mice. n=3.

Time (h)	Concentration of **ARC-18** (ng/mL)			Mean	SD
	No.1	No.2	No.3		
0.25	BLQ	BLQ	BLQ	NA	NA
0.5	1.50538	BLQ	BLQ	NA	NA
1	BLQ	BLQ	BLQ	NA	NA
2	BLQ	BLQ	2.29066	NA	NA
4	1.28795	1.77239	2.20122	1.75	0.46
6	BLQ	BLQ	BLQ	NA	NA
8	BLQ	BLQ	BLQ	NA	NA
24	BLQ	BLQ	BLQ	NA	NA

Time (h)	Concentration of **ATG** (ng/mL)			Mean	SD
	No.1	No.2	No.3		
0.25	5.95083	20.4308	BLQ	13.19	10.24
0.5	16.6284	23.4087	BLQ	20.02	4.79
1	21.3998	27.9299	BLQ	24.66	4.62
2	BLQ	BLQ	BLQ	NA	NA
4	BLQ	BLQ	BLQ	NA	NA
6	BLQ	3.40577	BLQ	NA	NA
8	BLQ	BLQ	BLQ	NA	NA
24	BLQ	BLQ	BLQ	NA	NA

Supplementary Table 2. Changes of drug concentration in the plasma after a single oral gavage of **ATG** (21.6 mg/kg, 0.058 mol) in rat. n=3.

Time (h)	Concentration of **ATG** (ng/mL)			Mean	SD
	No.1	No.2	No.3		
0.25	2.74	4.31	13.43	6.83	5.77
0.5	1.97	7.97	13.80	7.91	5.92
1	1.58	10.63	13.36	8.52	6.17
2	1.15	3.42	2.08	2.21	1.14
4	1.10	2.56	2.82	2.16	0.93
6	13.18	14.96	16.27	14.80	1.55
8	0.63	6.08	7.32	4.68	3.56
24	0.70	BLQ	6.56	3.63	4.14

Supplementary Table 3. Changes of PK parameter in the plasma after a single oral gavage of **ARC-18**(35 mg/kg, 0.058 mol) in rat. n=3.

**Compound ARC-18**	No.1	No.2	No.3	Mean	SD
$T_{1/2}$ (h)	NA	NA	NA	NA	NA
T_{max} (h)	0.5	4	2	2.17	1.76
C_{max} (ng/mL)	1.51	1.77	2.29	1.86	0.4
$AUC_{(0-t)}$ (h*ng/mL)	5.26	3.62	6.78	5.22	1.58
$AUC_{(0-\infty)}$ (h*ng/mL)	NA	NA	NA	NA	NA
$MRT_{(0-t)}$ (h)	2	3.21	2.65	2.62	0.6
$MRT_{(0-\infty)}$ (h)	NA	NA	NA	NA	NA

**Compound ATG**	No.1	No.2	No.3	Mean	SD
$T_{1/2}$ (h)	NA	NA	NA	NA	NA
T_{max} (h)	1	1	NA	1	NA
C_{max} (ng/mL)	21.40	27.93	NA	24.66	NA
$AUC_{(0-t)}$ (h*ng/mL)	13.07	99.21	NA	56.14	NA
$AUC_{(0-\infty)}$ (h*ng/mL)	NA	NA	NA	NA	NA
$MRT_{(0-t)}$ (h)	0.68	1.35	NA	1.01	NA
$MRT_{(0-\infty)}$ (h)	NA	NA	NA	NA	NA

Supplementary Table 4. Changes of PK parameter in the plasma after a single oral gavage of **ATG** (21.6 mg/kg, 0.058 mol) in rat. n=3.

**Compound ATG**	No.1	No.2	No.3	Mean	SD
$T_{1/2}$ (h)	NA	NA	NA	NA	NA
T_{max} (h)	6.00	6.00	6.00	6.00	0.00
C_{max} (ng/mL)	13.18	14.96	16.27	14.80	1.55
$AUC_{(0-t)}$ (h*ng/mL)	44.20	58.28	178.22	93.57	73.65
$AUC_{(0-\infty)}$ (h*ng/mL)	NA	NA	NA	NA	NA
$MRT_{(0-t)}$ (h)	7.98	4.61	11.36	7.98	3.37
$MRT_{(0-\infty)}$ (h)	NA	NA	NA	NA	NA
