# Supplementary material for: Arctigenin derivative (ARC-18) improved mitochondrial dysfunction and ameliorated frataxin deficiency symptoms via PGC-1α signaling
Source: Genes Dis. 2025 Sep 1;13(4):101838. doi: 10.1016/j.gendis.2025.101838 (PMC13011025; doi:10.1016/j.gendis.2025.101838)
Supplement: Multimedia component 3 [file mmc3.pdf]

Supplementary Figures

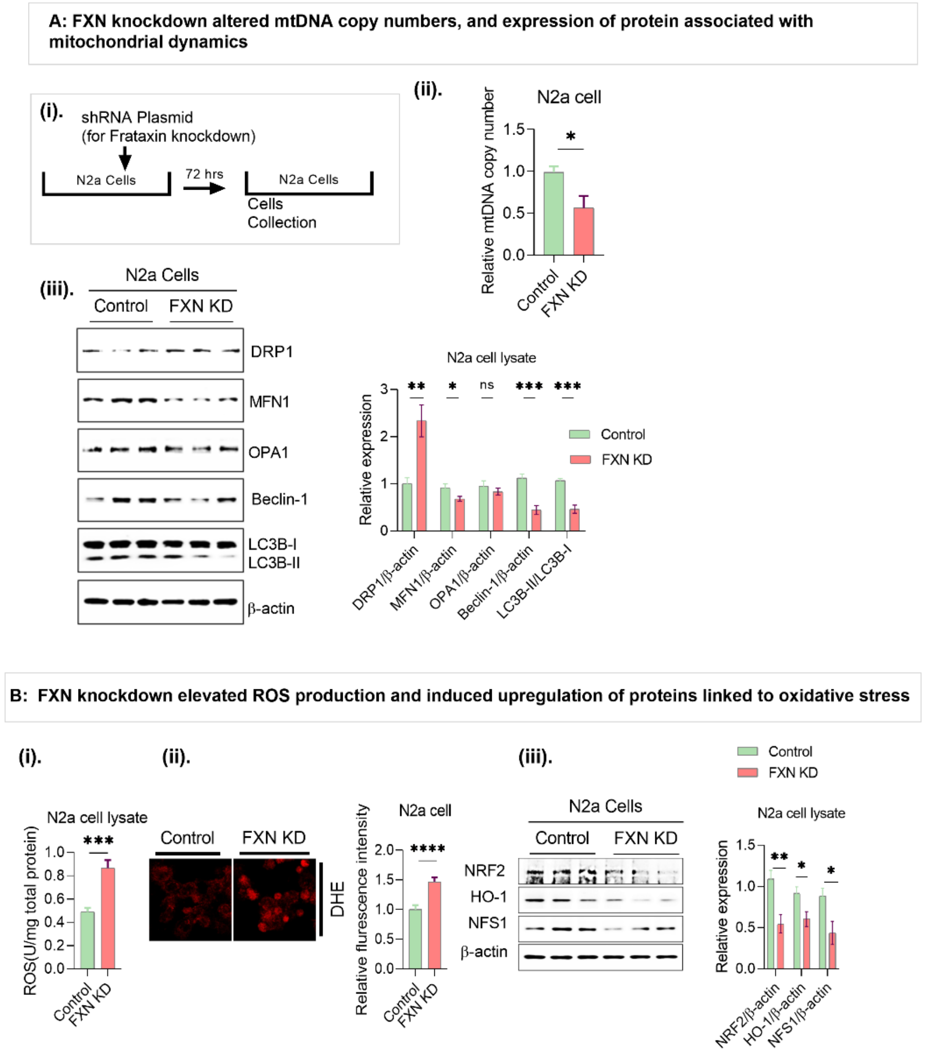

Figure S1: FXN downregulation altered mitochondrial and autophagy-related gene expression in N2a Cells.

A. (i) FXN expression was downregulated in N2a cells using a shRNA plasmid. (ii) Bar graphs illustrate changes in relative mtDNA number (n=4). (iii) Representative immunoblot images and bar graphs display the expression levels of mitochondrial and autophagy-related genes, including DRP1, MFN1, OPA1, Beclin-1, LC3B, and β-actin as a loading control. n=5-6

B. (i) ROS levels (n=7-10) in the N2a cells. (ii) DHE staining and its quantitative bar graph quantify ROS production. n=30. (iii) The Representative immunoblot images and bar graphs display the expression levels of mitochondrial and autophagy-related genes, including NRF2, HO-1, NFS1, and β-actin as a loading control. n=5-6.

Data are presented as mean ± SEM and analyzed using Student's t-test, one-way ANOVA, and post hoc analysis. Differences were considered significant at $p < 0.05$ (*), $p < 0.01$ (**), $p < 0.001$ (***), or $p < 0.0001$ (****). ns indicates non-significance.

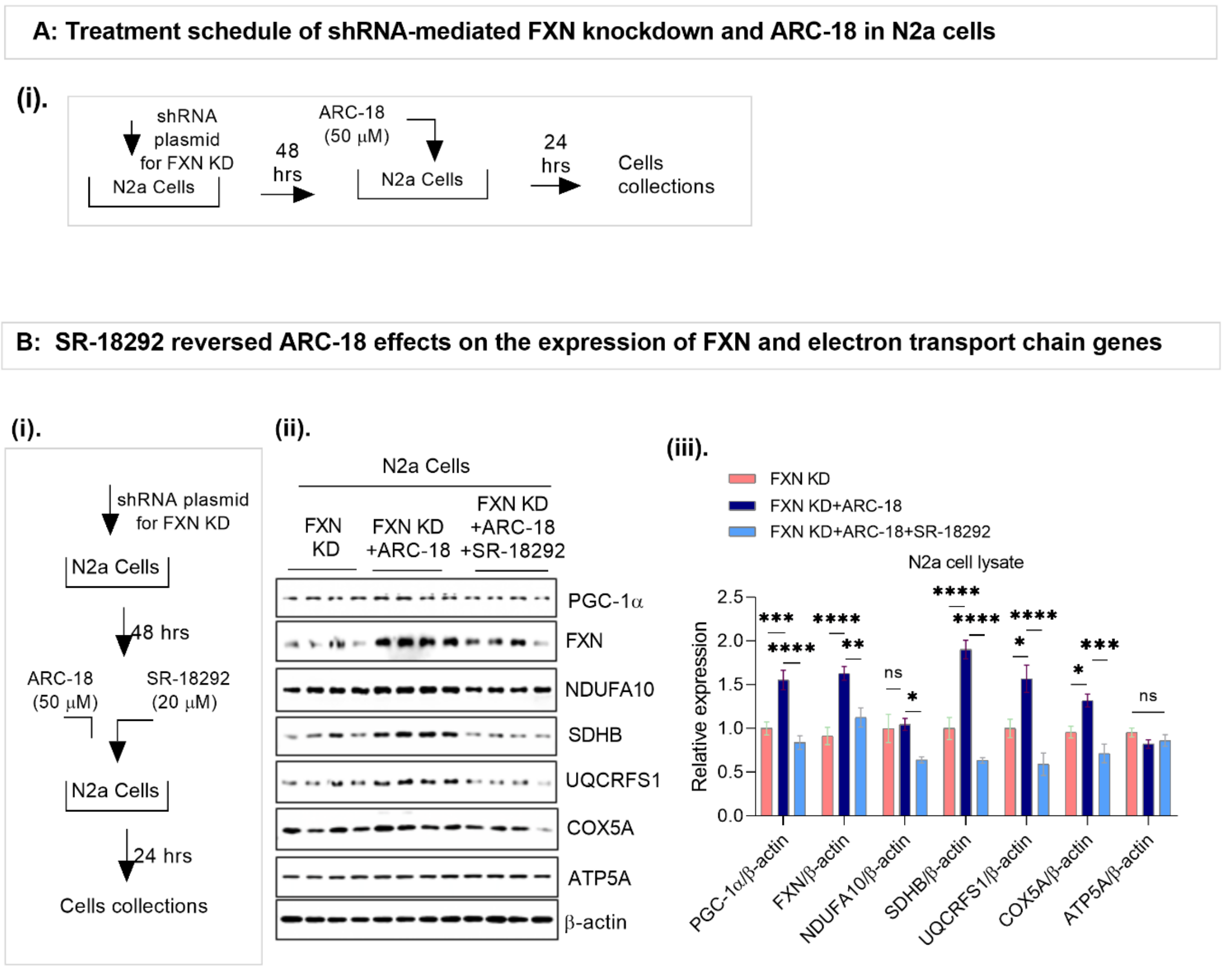

Figure S2: SR-18292 abated ARC-18 effects.

A. (i) ARC-18 treatment schedule to N2a cells.

B. (i) SR-18292 was administered to N2a cells previously treated with ARC-18 and FXN downregulation. (ii) Immunoblot analysis and bar graphs show the expression levels of mitochondrial genes, including PGC-1α, FXN, NDUFA10, SDHB, UQCRFS1, ATP5A, and β-actin (loading control), in the SR-18292-treated FXN-deficient ARC-18-treated N2a cells. n=4 for NDUFA10, and n=8-9 for others.

Data are presented as mean ± SEM and analyzed using Student's t-test, one-way ANOVA, and post hoc analysis. Differences were considered significant at $p < 0.05$ (*), $p < 0.01$ (**), $p < 0.001$ (***), or $p < 0.0001$ (****). ns indicates non-significance.

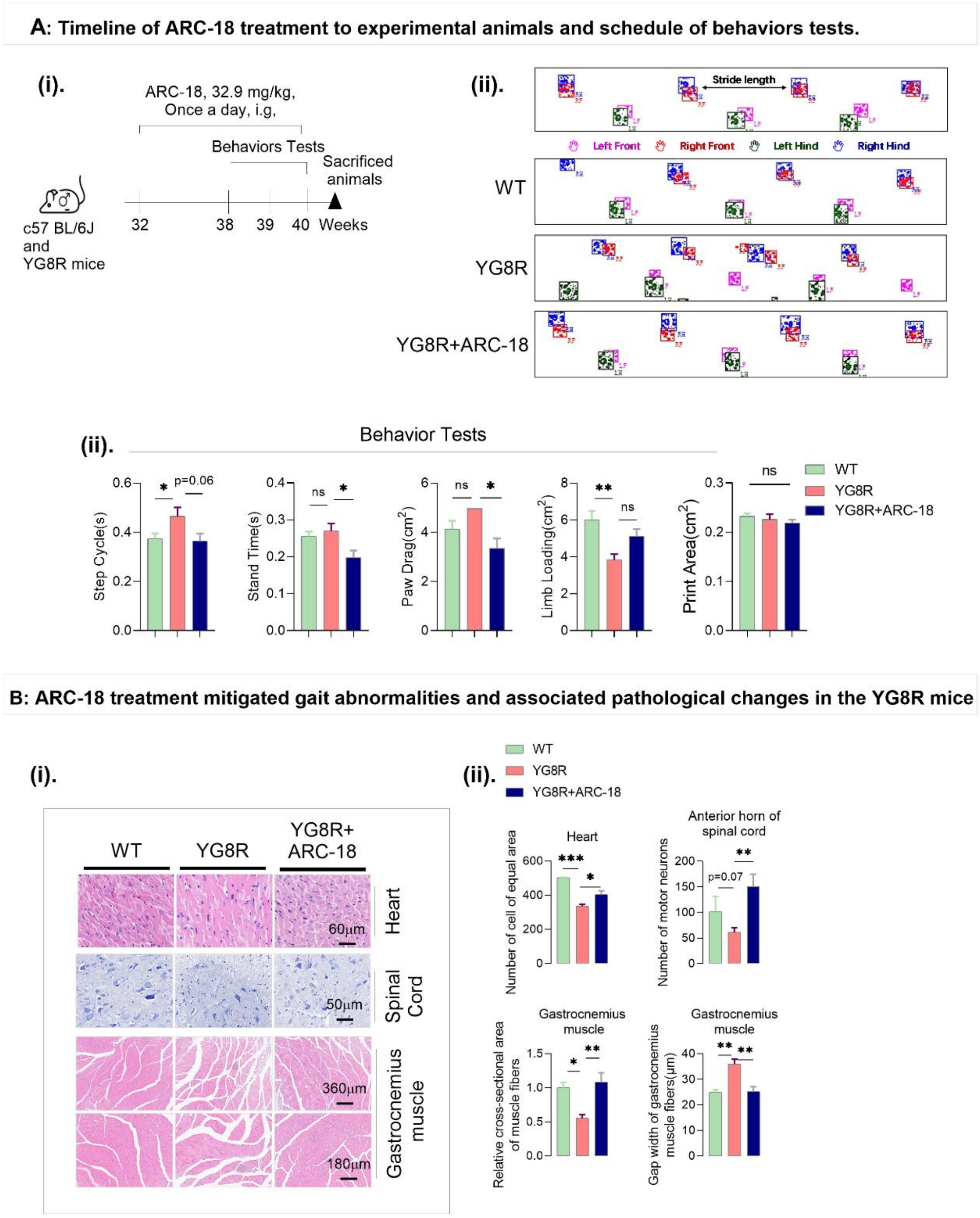

Figure S3: ARC-18 ameliorates motor function deficits and histological abnormalities in YG8R mice.

A. ARC-18 treatment schedule/timeline. Gait analysis revealed that ARC-18 treatment significantly restored gait parameters in YG8R mice. n=7-15.

B. ARC-18 treatment significantly improved histological abnormalities in YG8R mice, as evidenced by increased cell number in the heart (H&E staining), improved motor neurons in the spinal cord (Nissl staining), and increased cross-sectional area of muscle fibers in the gastrocnemius muscle (H&E staining), while decreasing gap width of muscle fibers in the gastrocnemius muscle (H&E staining). n=3-4.

Data are presented as mean ± SEM and analyzed using one-way ANOVA and post hoc analysis. Differences were considered significant at $p < 0.05$ (*), $p < 0.01$ (**), $p < 0.001$ (***), or $p < 0.0001$ (****). ns indicates non-significance.

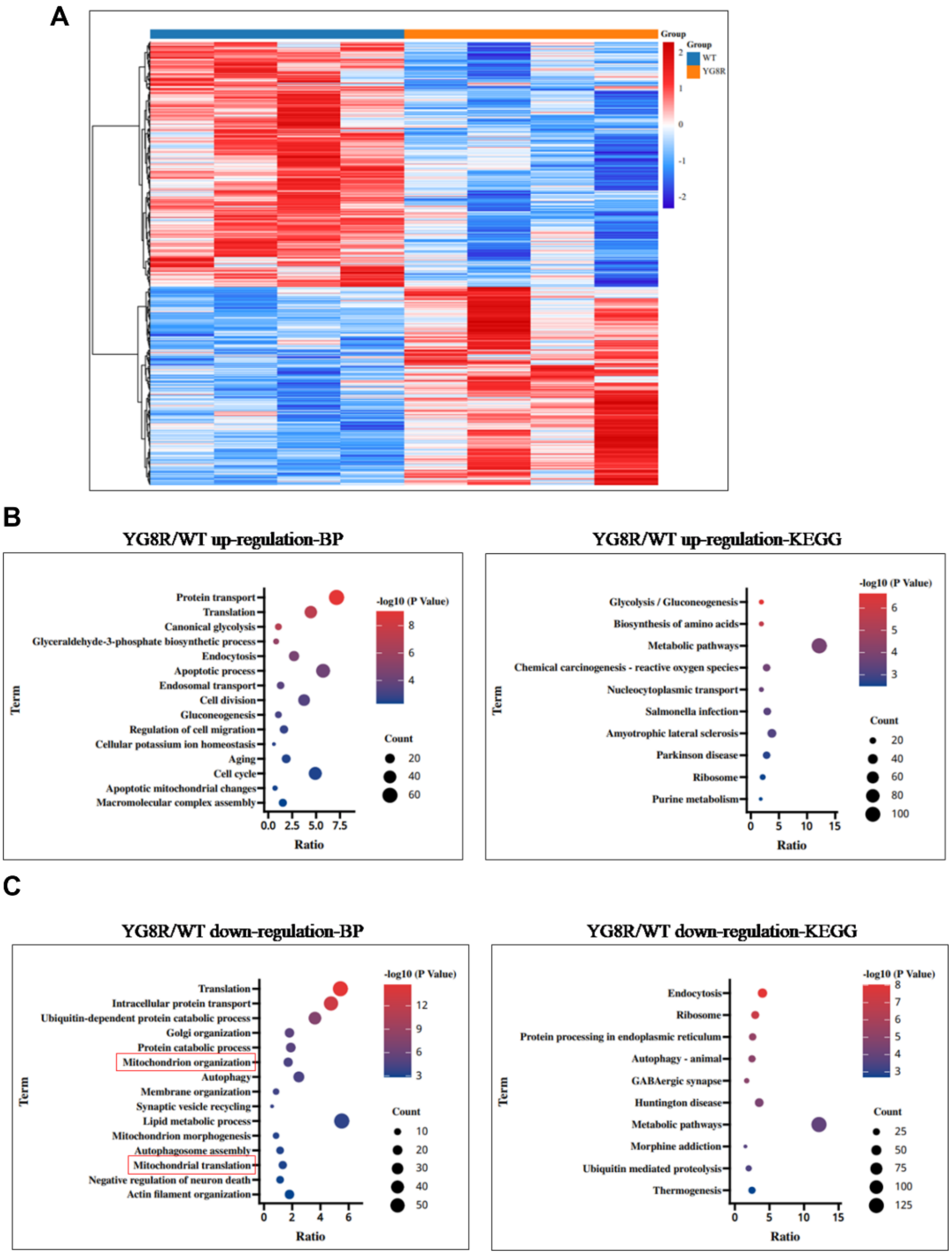

Figure S4: Proteomic analysis of cerebellum tissue in WT and YG8R mice.

A. Heat map shows upregulated and down-regulated differential proteins in these two groups. n=4.

B. Biological processes and KEGG pathways involving the upregulated differentially expressed proteins.

C. Biological processes and KEGG pathways involving the downregulated differentially expressed proteins.

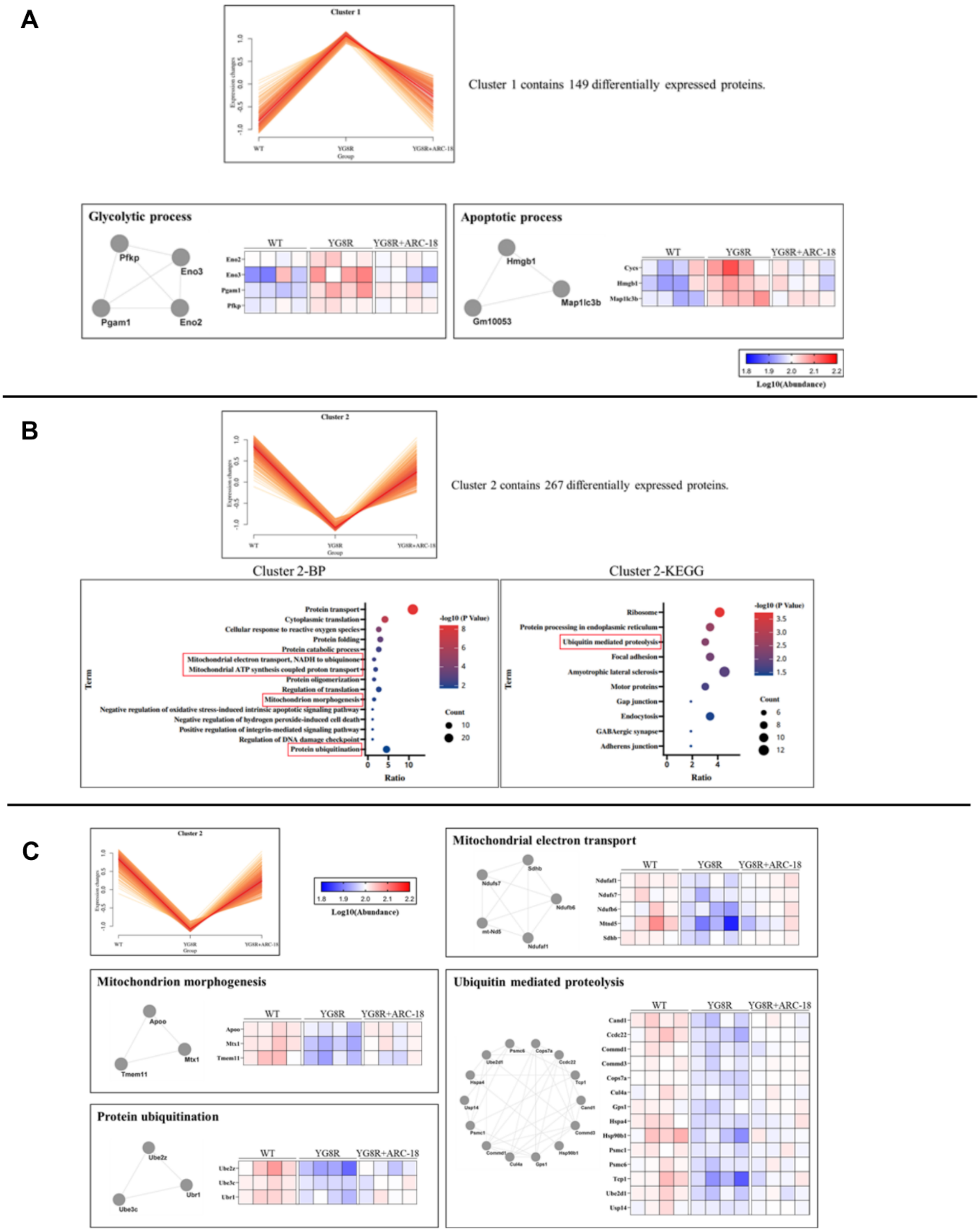

Figure S5: Molecular complex assay (MCODE) analysis demonstrated the potential protein interaction networks and trends of relevant differential proteins.

A. Prediction of interactions of proteins within the pathway with significant changes in the cluster 1 of proteins.

B. Biological processes and KEGG pathways involving the differentially expressed proteins in cluster 2.

C. Prediction of interactions of proteins within the pathway with significant changes in the cluster 2 of proteins.

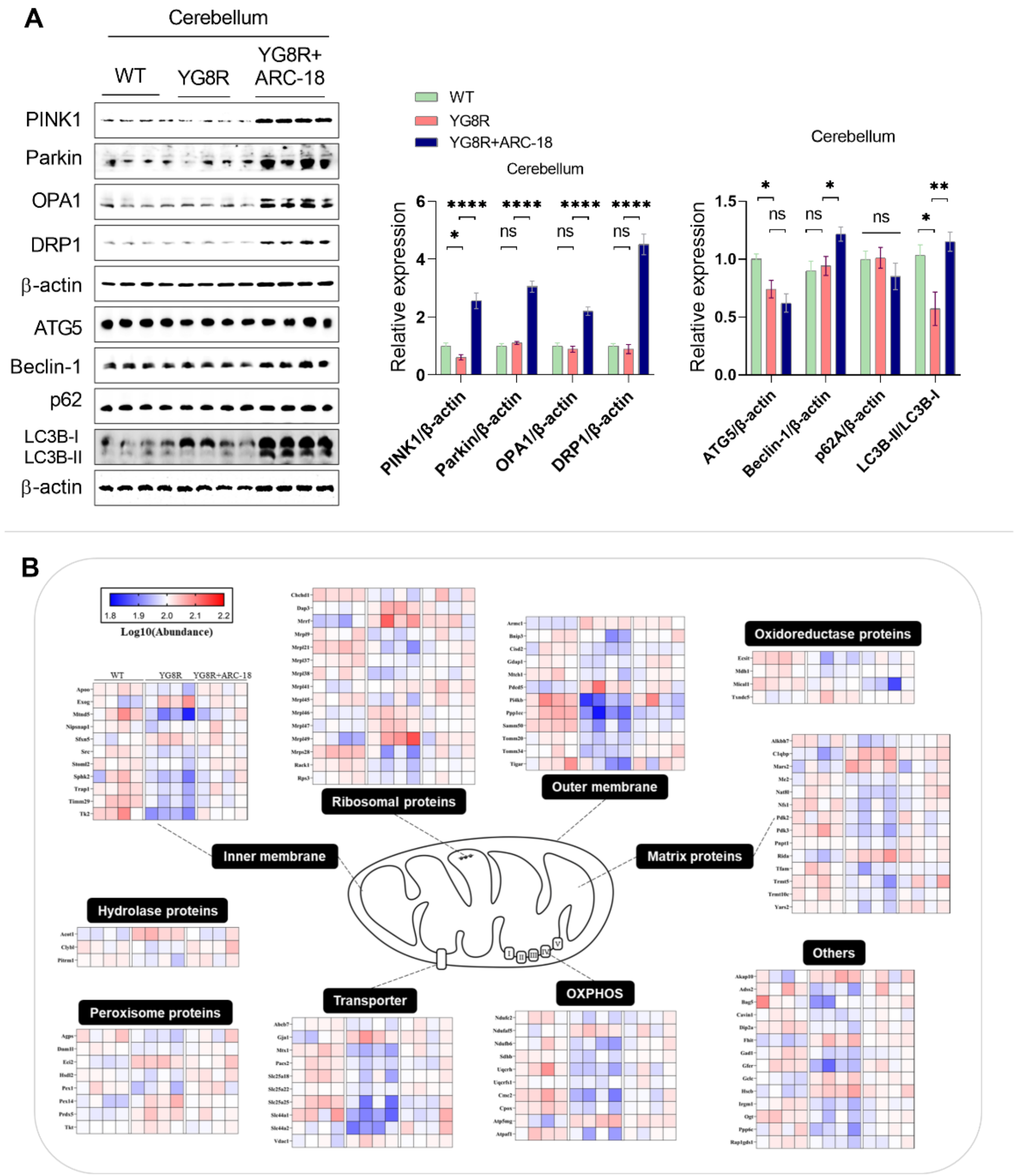

Figure S6: ARC-18 treatment improved mitochondrial and autophagy defects in the YG8R mice cerebellum.

A. Representative immunoblot images and bar graphs show the expression levels of mitochondrial and autophagy-related genes, including PINK1, Parkin, OPA1, DRP1, ATG5, Beclin-1, P62, LC3B, and β-actin as a loading control. n=6-8.

B. Mitochondrial protein expression map of WT/YG8R/YG8R+ARC-18 groups with significant changes.

Data were expressed as mean ±SEM and analyzed via One-way ANOVA and posthoc analysis. p= <0.05 was considered significant. (*): p <0.05, (**): p <0.01, (***): p <0.001. ns= nonsignificant.

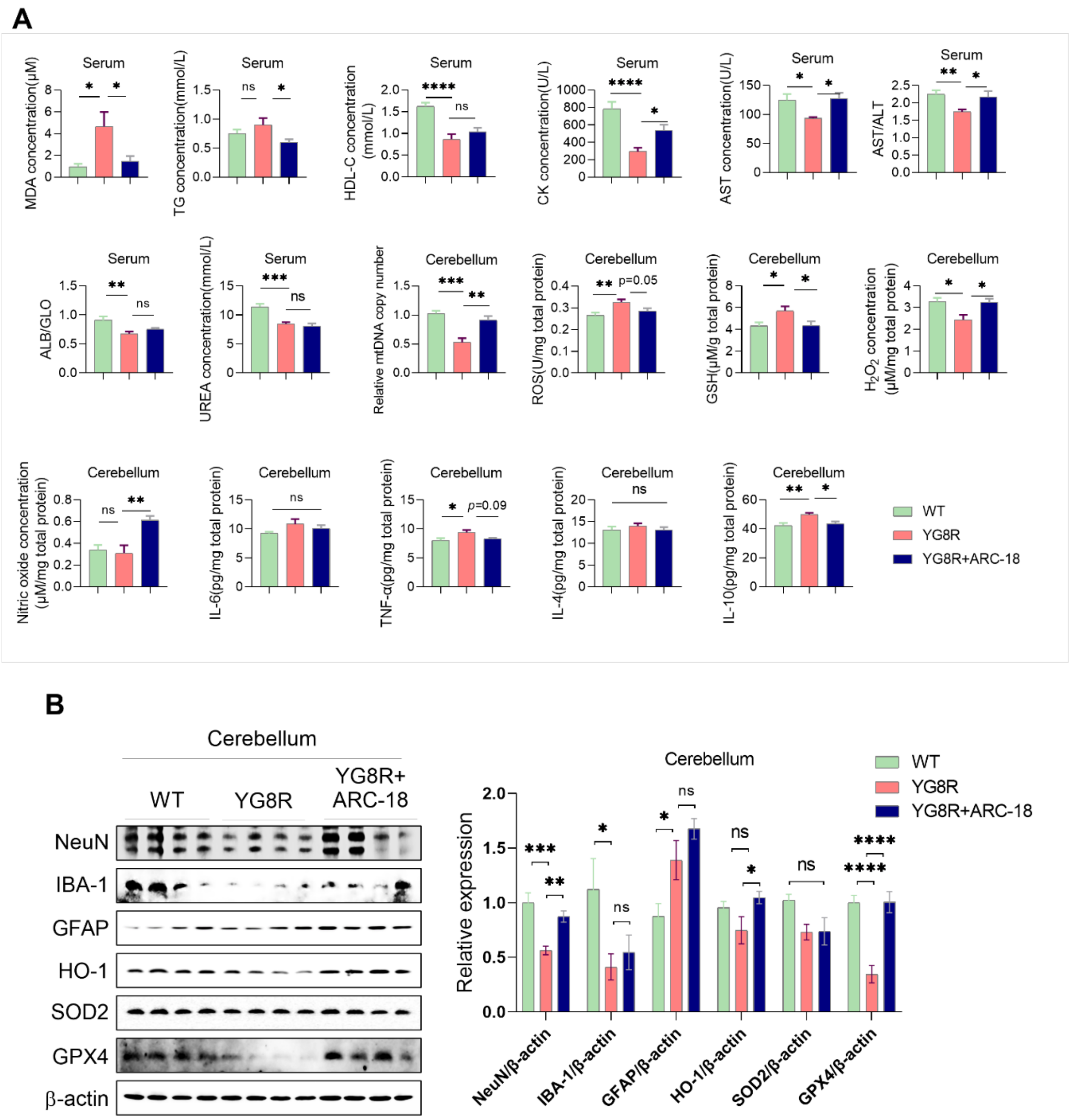

Figure S7: ARC-18 improves oxidative and inflammatory-related impairment in the serum and cerebellum of the YG8R mice.

A. Bar graphs show Serum MDA, TG, HDL-C, CK, AST, AST/ALT ratio, ALB/GLO ratio, and UREA concentration. n=5-10.

B. Bar graphs show the concentration/level of mtDNA, ROS, GSH, H2O2, NO, IL-6, TNF-α, IL-4, and IL-10. n=3-4 for mtDNA, n=6-8 for others.

C. Representative immunoblot images and bar graphs show the expression levels of NeuN, IBA-1, GFAP, HO-1, SOD2, GPX4, and β-actin as a loading control. n=6-8

Data were expressed as mean ±SEM and analyzed via One-way ANOVA and posthoc analysis. p= <0.05 was considered significant. (*): p <0.05, (**): p <0.01, (***): p <0.001, (****): p <0.0001. ns= nonsignificant.

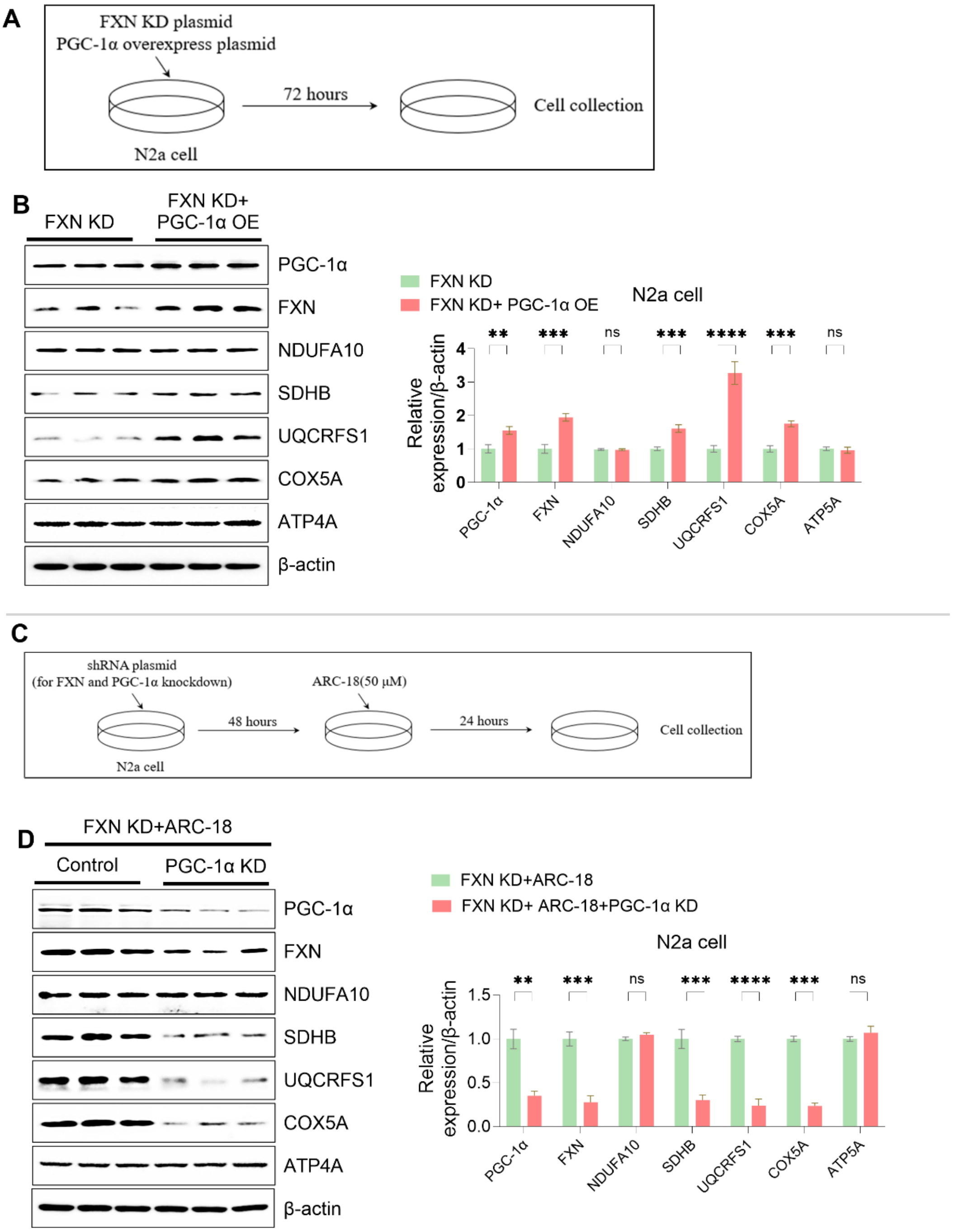

Figure S8: PGC-1α Overexpression and Knockdown Modulate Expression of Mitochondrial Complex Subunits in FXN-Deficient N2a Cells

A. Schematic of experimental design for PGC-1α overexpression in FXN knockdown (FXN KD) N2a cells. Cells were transfected with FXN KD and PGC-1α overexpression plasmids and harvested after 72 hours.

B. Representative Western blots and quantification showing expression levels of PGC-1α, FXN, and mitochondrial electron transport chain complex subunits (NDUFA10, SDHB, UQCRFS1, COX5A, ATP4A) in FXN KD cells with or without PGC-1α overexpression (OE). Quantification of relative expression normalized to β-actin is shown (n=3-6); $^{**}p < 0.01$, $^{***}p < 0.001$, $^{****}p < 0.0001$, ns = not significant).

C. Experimental schematic for dual knockdown (FXN and PGC-1α) in the presence of ARC-18 treatment.

N2a cells were transfected with shRNA plasmids targeting FXN and PGC-1α for 48 hours, then treated with ARC-18 (50 µM) for 24 hours before collection.

D. Representative Western blots and quantification of PGC-1α, FXN, and mitochondrial complex subunits in FXN KD+ARC-18 treated cells with or without PGC-1α knockdown. Quantification shows relative expression to β-actin (n=3-6); **p < 0.01, ***p < 0.001, ****p < 0.0001, ns = not significant).

Data were expressed as mean ±SEM and analyzed via One-way ANOVA and posthoc analysis. *p*= <0.05 was considered significant. (*): p <0.05, (**): p <0.01, (***): p <0.001, (****): p <0.0001. ns= nonsignificant.
