# Supplementary material for: Arctigenin derivative (ARC-18) improved mitochondrial dysfunction and ameliorated frataxin deficiency symptoms via PGC-1α signaling
Source: Genes Dis. 2025 Sep 1;13(4):101838. doi: 10.1016/j.gendis.2025.101838 (PMC13011025; doi:10.1016/j.gendis.2025.101838)
Supplement: Multimedia component 4 [file mmc4.pdf]

Supplementary data (detailed Methods and Results)

Materials and Methods

Animals

Male YG8R mice (B6.Cg-Fxntm1Mkn Tg (FXN)YG8Pook/J, Stock No: 012253) were obtained from The Jackson Laboratory. These mice gradually developed a progressive Friedreich ataxia phenotype as they aged. Wild-type (WT) C57BL/6J mice were purchased from Guangdong Medical Laboratory Animal Center as the controls suggested by Jackson Laboratory (https://www.jax.org/strain/012253). All mice were housed under controlled environmental conditions with a 12-hour light/dark cycle and a temperature range of 18-22°C. They were provided with standard rodent chow and tap water ad libitum throughout the study. All experimental procedures and protocols were approved by the Institutional Animal Care and Use Committee of Peking University Shenzhen Graduate School (Approval number: 11110).

All experiments used mice at 8 months of age. Behavioral testing was conducted over 2 months, after which the animals were sacrificed at 10 months of age.

Drug Treatment and Tissue Collection

The State Key Laboratory of Biotherapy at Sichuan University synthesized and provided ARC-18, a novel derivative of arctigenin with better water solubility and bioavailability. The detailed synthesis procedures and pharmacokinetic parameters can be found in the supplementary data. The dosage of ARC-18 used in the experiments was converted from an equivalent molar dose of Arctigenin in mouse studies and dissolved in purified water. [1].

The drug treatment group was administered ARC-18 orally at a dose of 32.9 mg/kg once daily for eight weeks (Figure S3). The control group received an equivalent volume of purified water. Behavioral tests were conducted during the final two weeks of the treatment period. Cerebellum, spinal cord, heart,

gastrocnemius muscle, and serum samples were collected and stored at -80°C for subsequent analysis.

Behavior Analysis

Pole Test

Motor and coordination abilities were assessed using a pole test [2]. A wooden pole (50 cm in length, 1 cm in diameter) was vertically fixed on a base and wrapped with gauze to increase friction. Mice were trained to climb down the pole independently (once daily for two consecutive days). During the test, mice were placed at the top of the pole, and the time to fully descend was recorded. Each mouse performed three trials with a 5-minute rest interval between trials to avoid fatigue. The average climbing time was used as the evaluation metric.

Beam Walking Test

Motor and coordination abilities were assessed using a beam walk test [3]. A wooden beam (100 cm length, 12 cm width) was horizontally fixed 20 cm above the foam-padded surface. Mice were trained to walk across the beam independently, and the time taken to traverse the beam was recorded. The procedure was repeated three times for each mouse, and the average passage time was used as the evaluation metric.

Grip Strength Test

Grip strength was assessed using a grip strength test [2]A mouse grip tester (YLS-13A, Nuolei Xinda Technology Co., LTD, China) was used to quantitatively evaluate the grip strength of mice. Each mouse was placed in the center of a grip plate, and its tail was gently pulled downward to encourage grasping. Once the mouse firmly grasped the plate, its tail was pulled backwards until it released its grip. This procedure was repeated five times for each mouse, and the maximum grip strength recorded was used as the

evaluation metric.

Wire Hanging Test

Wire-hanging test was used to evaluate the strength intensity of mice [2]. Gently placed each mouse on a horizontal wire mesh, and slowly inverted the mesh and raised it to a height of 50 cm above the ground after its front paw firmly grasped it. A soft foam pad was placed below it to prevent injury when the mouse fell. The experimental process was repeated three times, with a minimum interval of 15 minutes between each two to prevent mouse fatigue. The latency period of each mouse's landing was recorded as the evaluation value.

Gait Analysis

A gait analysis system (BT60601, Stones Scientific Instruments, China) was used to evaluate the gait of autonomous movement in mice. The system used high-speed digital cameras to record the steps of mice and automatically analyzed various gait parameters. The experiment was conducted in a dark environment, and 3-5 consecutive steps were recorded for the mice. After each animal experiment, I wiped and cleaned the walking path with 75% ethanol to avoid interference from previous animal odor. Gait characteristics are described using parameters such as stride length, swing speed, step cycle, etc.

Histological Analysis

After anaesthetizing the mice with pentobarbital sodium (n=3-4), we performed cardiac perfusion with PBS buffer, quickly removed the complete mouse cerebellum, spinal cord, heart, and gastrocnemius muscle tissues, and soaked them in a 4% paraformaldehyde solution at 4 ℃ for 48 hours. Next, dehydrated them with gradient ethanol, placed the tissues in an embedding box, and embedded them in paraffin. A rotary slicer (RM2235, Leica, Germany) was used

to slice the tissue into 5 μm sections attached to glass slides, dried, and stored at room temperature. Then, the following staining methods were performed, and digital images of the slices were obtained through a scanning microscope imaging system, followed by corresponding analysis [4].

H&E Staining

A Hematoxylin eosin staining kit (G1120, Solarbio, China) was used to optimize the conditions according to the instructions of the reagent manufacturer. Cerebellum, heart and gastrocnemius muscle sections were stained in the following order: dewaxed with xylene(10 mins × 2)and gradient ethanol to water(90%, 75%, 50%, 2 mins for each), stained with hematoxylin for 10 mins, washed with distilled water(2 secs) and differentiation(30 secs), stained with eosin for 2 mins, dehydration with gradient ethanol after washing(50%, 75%, 90%, 100%, 10 secs for each), xylene transparency(10 mins×2) and finally sealing with neutral gum. [2].

Nissl Staining

A Nissl staining kit (G1430, Solarbio, China) was used to optimize the conditions according to the instructions of the reagent manufacturer. Cerebellum and L4-L5 spinal cord sections were stained in the following order: dewaxed with xylene(10 mins×2)and gradient ethanol to water(90%, 75%, 50%, 2 mins for each), soaked in Nissl staining solution for 45 mins at 60 °C, washed with distilled water, dehydration with gradient ethanol(50%, 75%, 90%, 100%, 10 secs for each) and xylene transparency(10 mins×2), and finally sealing with neutral gum [5].

Prussian Blue Iron Staining

A Prussian Blue Iron Stain Kit (G1422, Solarbio, China) was used to optimize the conditions according to the instructions of the reagent manufacturer. Cerebellum sections were stained in the following order: dewaxed with

xylene(10 mins×2)and gradient ethanol to water(90%, 75%, 50%, 2 mins for each), soaked in Perls solution for 20 mins at 37 °C, washed with distilled water, dyeing working solution and enhancement solution for 10 mins, washed with distilled water, dehydration with gradient ethanol(50%, 75%, 90%, 100%, 10 secs for each), xylene transparency(10 mins×2) and finally sealing with neutral gum.

Proteomics

Sample Preparation

Cerebellum tissues from each group of animals (n=4) were placed in PBS solutions containing 8M urea, protease, and phosphatase inhibitors. In the second place, lysed the tissues by using low-temperature ultrasound (VCX-150, Sonics, USA). After the samples were lysed and centrifuged at 4 ℃ and 12000 × g for half an hour, the upper clear liquid was taken for BCA assay (P0011, Beyotime, China). 25 μg of protein was taken from each sample and added dithiothreitol (DTT, final concentration 10 mM). Next, Mixed well and incubated at 55 ℃ for 1 hour, incubated with iodoacetamide (IAA, final concentration 25 mM) at room temperature for 1 hour, preventing from the light.

Then, the pancreatic enzyme (1:25w/w) (V5072, Promega, USA) was added to the sample and digested at 37 ° C for 4 hours. After this, diluted the urea concentration in the sample to 1M using PBS (pH=8.0) and continued to digest at 37 °C for 11 hours. After digestion, the pH of the solution was adjusted to 1-2 with trifluoroacetic acid (TFA), desalinating it in a desalination column (186000383, Waters, USA) before drying at a low temperature.

TMT Labeling and LC-MS/MS Analysis

The dried sample was re-dissolved in 50 μL 200mM triethylammonium bicarbonate (TEAB) and then labelled with TMT reagent at room temperature for 1 hour. The reaction was terminated with 5% hydroxylamine. Then, mixed samples with different labels were desalinated through a desalination column,

dried at a low temperature, and dissolved again in 100 μL of 0.1% formic acid. Then, the labelled peptide was added to the chromatography column (WAT094225, Waters, USA) and separated the peptide sample into 60 components using Extreme 3000 UHPLC (Thermo Fisher Science, USA). All components were combined into 15 tubes by centrifugation and low-temperature drying and dissolved in 30 μ L of 0.1% formic acid. After vortex centrifugation, the upper clear liquid was taken and passed through Liquid chromatography (LC) mass spectrometry (MS)/MS analysis.

Finally, proteomics discovery software was used to calculate the relative expression levels of each protein in the mouse database based on the reported ion strength of each peptide. Using the t-test method in Perseus software, the differential expression of proteins in the cerebellum tissues of mice in the model group and control group, as well as the model group and ARC-18 treatment group, was set to a p-value less than 0.05.

Bioinformatics Analysis

Heatmaps and David's bioinformatics resources was used to analyze the relative abundance of differentially expressed proteins in each group (https://david.ncifcrf.gov/). Cluster analysis was performed to demonstrate corresponding biological processes and KEGG enrichment pathways. We also used Molecular Complex Detection (MCODE) to find dense regions of protein-protein interaction (PPI), and finally visualized images by Hiplot (https://hiplot-academic.com/).

ELISA

The frozen samples were ultrasonically homogenized in cold RIPA buffer (P0013B, Beyotime, China) supplemented with protease inhibitors (20124ES, Yisheng Biotechnology, China). The homogenates were then centrifuged at 12,000×g for 15 mins at 4°C. Protein concentrations were quantified by using

the BCA assay kit (P0011, Beyotime, China). ELISA experiments were performed according to the manufacturer's instructions by using the reagents provided (RK04770, Abclonal, China). Specifically, a pre-coated 96-well plate containing capture antibodies was used to generate a six-point standard curve by serial dilution of the corresponding standard product. A total of 20 μL of each sample and an equivalent volume of diluent were added to the wells as a negative control. The plate was incubated at 37°C for 2 hours. Following incubation, the liquid was discarded, and the wells were washed three times with the wash buffer provided in the kit. Subsequently, 100 μL of biotinylated detection antibody was added, and the plate was incubated at 37°C for 1 hour. After discarding the liquid and washing the wells again, streptavidin-HRP working solution was added and incubated at room temperature for 30 minutes. TMB substrate solution was added, and the reaction was allowed to proceed for 15 minutes in the dark before being terminated with the stop solution. The absorbance of the liquid in each well was measured at 450 nm within 15 minutes using a microplate reader. The concentration of the target analyte in each well was calculated based on the standard curve and normalized to the protein concentration for statistical analysis.

Mitochondrial Copy Number Detection

After extracting genomic DNA from cerebellum tissue and cell samples using the TIANamp Genomic DNA kit (DP304, TIANGEN, China), qPCR detection was performed using TransScript Tip Green qPCR SuperMix (AQ601, Transgen Biotech, China). Relative abundance was normalized to nuclear control.

Primer sequences for the mitochondrial segment:

(F) GCCAGCCTGACCCATAGCCATAAT

(R) GCCGGCTGCGTATTCTACGTTA.

Primer sequences for the single-copy nuclear control:

(F) TTGAGACTGTGATTGGCAATGCCT

(R)CCAGAAATGCTGGGCGCTCACT.

Cell culture and treatment

Mouse neuroblastoma N2a cell line was cultured in DMEM medium (C11995500BT, Gibco, USA) containing 10% fetal bovine serum in a humidified incubator at 37 °C and 5% CO_2. The dosage of ARC-18 treatment was selected using the cell counting kit-8 (HY-K0301, MedChemExpress, USA), and the treatment time was based on previous studies [6]. The processing conditions for SR-18292 are also based on prior research [7].

The plasmid was used in the experiment to knock down Frataxin (TG514670, Origene, USA). According to the manufacturer's instructions, Lipofectamine 3000 (L3000015, Thermo, USA) was used for transfection, and cells were collected 72 hours later. Among the four different sequences of shRNA supplied by the manufacturer, we verified that only shRNA-A had a significant knockdown effect and conducted experiments based on the control plasmid and shRNA-A. The detailed sequence is as follows:

shRNA-A: ACGAGACAGCGTATGAAAGACTGGCGGAA;

shRNA-B: TCGTAACCTGCGGCCGCCGAGGCCTACAT;

shRNA-C: AAACAAGCAAATCTGGCTGTCTTCTCCTT;

shRNA-D: AACACCAAACTGGACTTGTCTTCATTGGC.

For the PGC-1α overexpression and knockdown experiments, we used a plasmid provided by Shenzhen Yanming Biotechnology Co., Ltd. The transfection protocol followed the same timeline as the FXN knockdown plasmid. The overexpression plasmid contained the full-length mouse PGC-1α gene sequence, while the knockdown plasmid was designed to silence PGC-1α expression effectively.
(Plasmid sequence:
AATTGGGAACAGCAGCAGAGACAAATCTCGAGATTTGTCTCTGCTGCTGTTCCTTTTTT)

Reactive oxygen species probe detection

The ROS probe employed in the experiment (CA1420, Solarbio, China) was

utilized following the manufacturer's instructions. Briefly, N2a cells were inoculated in a small dish appropriate for microscopy, transfected with the plasmid(shRNA-A) and waited for 72 hours. Subsequently, the medium was removed, washed with PBS, and the probe diluted with serum-free medium (1:1000) was added and incubated at 37℃ for 30 minutes. The dye was washed three times with PBS and then observed under a confocal laser microscope.

Western Blot

Cerebellum and cell sample lysis and protein quantification were as described above. The supernatant was mixed with the loading buffer and boiled at 100°C for 10 minutes. After denaturation, the samples were added in the SDS-PAGE gel, which was prepared in advance, and the electrophoresis system (Bio-Rad, USA) was used for separation. After the protein was separated by molecular weight, the protein was horizontally transferred to the nitrate cellulose membrane (66485, Pall, USA) by electrophoresis. At room temperature, the membrane was closed with TBST (tris buffered brine, 0.1% Tween 20) containing 5% skim milk powder for 1 hour to prevent non-specific binding. After closure, the membrane was washed three times with TBST, cut according to the molecular weight of the desired target protein, and incubated at 4℃ overnight. The next day, the primary antibody was recovered, the membrane was cleaned with TBST, and the secondary antibody (anti-rabbit or anti-mouse, depending on the primary antibody) coupled with horseradish peroxidase (HRP) was incubated. The protein bands were imaged with the Bio-Rad ChemiDoc XRS+ imaging system (Bio-Rad Laboratories) after TBST was washed several times. Density analysis of protein bands was performed using ImageJ software (National Institutes of Health, Bethesda, USA). Band strength was normalized to β-actin or total protein [8,9,2].

Statistical Analysis

Western blot band intensities and morphological data were quantified using ImageJ (version 1.30) and GraphPad Prism 8. Statistical analysis was performed using one-way ANOVA followed by Tukey's multiple comparison test. Data are presented as mean ± standard error of the mean (SEM). Differences were considered statistically significant at *$p < 0.05$, **$p < 0.01$, ***$p < 0.001$, and ****$p < 0.0001$.

Results

Frataxin Deficiency Impaired Mitochondrial Dynamics and Biogenesis in N2a Cells

Frataxin (FXN) deficiency is a central driver of mitochondrial dysfunction in Friedreich’s ataxia (FA). In this study, shRNA-mediated FXN knockdown in N2a cells (Figure S1A, i) successfully reduced FXN protein levels (Figure 1a, b), triggering mitochondrial DNA depletion and elevated reactive oxygen species (ROS) (Figure S1A, and B). The deficiency disrupted mitochondrial dynamics, evidenced by increased DRP1 (fission) and decreased MFN1/OPA1 (fusion) expression. Concurrently, impaired mitochondrial biogenesis and autophagy were observed through reduced Beclin-1 and LC3B-II levels. Critical mitochondrial regulators, including NRF2, HO-1, NFS1, AMPK1α, PGC-1α, SDHB, UQCRFS1, and COX5A—showed significant downregulation in FXN-deficient cells, while NDUFA10 and ATP5A remained unchanged (Figure 1a, b; Figure S1A, iii). These in vitro findings were corroborated by proteomic analysis of the YG8R mouse cerebellum, which revealed parallel pathway alterations (Figures S4). These results demonstrate the essential role of FXN in maintaining mitochondrial homeostasis through the regulation of dynamics, biogenesis, and autophagy, providing mechanistic insights into FA pathology.

ARC-18 improved Mitochondrial Dysfunction in FXN-deficient cells via PGC-1α pathway

PGC-1α, a pivotal regulator of mitochondrial biogenesis, was targeted in this study. Arctigenin (ATG) was chemically modified, resulting in a novel ATG derivative, ARC-18 (detailed synthesis in Methods and Supplementary Data). FXN-deficient N2a cells were treated with ARC-18 for 24 hours (Figure S2A i). Comparative quantification of plasma ARC-18 and ATG concentrations (Supplementary Data) revealed a prolonged metabolic half-life for ARC-18. Notably, ARC-18 treatment of FXN-KD cells led to a significant upregulation of PGC-1α, FXN, SDHB, COX5A, and UQCRFS1 levels compared to untreated FXN-KD cells, while NDUFA10 and ATP5A levels remained unaffected (Figure 1 c and d). To further investigate the underlying mechanism and confirm PGC-1α role in ARC-18 effects, FXN-KD cells were co-treated with ARC-18 and the PGC-1α inhibitor SR-18292. As anticipated, SR-18292 effectively reversed the activating effects of ARC-18 on PGC-1α, FXN, NDUFA10, SDHB, UQCRFS1, and COX5A levels (Figure S2B). These results strongly indicate that ARC-18 enhances mitochondrial protein expression through a PGC-1α-dependent pathway.

ARC-18 Ameliorated Motor Dysfunction and Neurodegeneration in the YG8R Mice

To investigate ARC-18's effects on FXN deficiency in a living system, the YG8R mouse model was used. These mice received oral administration of ARC-18 for 8 weeks, after which behavioral assessments were conducted (Figure S3A). ARC-18 treatment significantly improved motor coordination, as demonstrated by reduced climbing time and beam walk duration, increased grip strength, and prolonged falling latency in the wire hanging test. Furthermore, YG8R mice treated with ARC-18 showed enhanced gait parameters, including increased stride length, body speed, swing time, and decreased stand time and paw drag.

However, ARC-18 did not affect limb loading, print area, or step cycle (Figure S3B). Histological analysis of the cerebellum revealed that ARC-18 treatment significantly increased the number of Purkinje cells and decreased the presence of iron-laden Purkinje cells in YG8R mice (Figure 1i and j).

Additionally, ARC-18 increased the granule cell population in the cerebellum. In the heart, ARC-18 reduced the occurrence of cells with uniform area, and in the spinal cord, it increased the number of motor neurons in YG8R mice. Moreover, ARC-18 alleviated muscle fiber atrophy and widened the gap width in the gastrocnemius muscle of YG8R mice (Figure S3B).

ARC-18 Modulated Mitochondrial Dynamics and Autophagy in YG8R mice cerebullum

To further elucidate the mechanisms by which ARC-18 exerts its protective effects, the effects of ARC-18 on mitochondrial and autophagy-related gene expression were examined. FXN deficiency led to significant alterations in the expression of proteins involved in mitochondrial function and autophagy, including PINK1, PGC-1α, AMPKα, and LC3B. Remarkably, treatment with ARC-18 reversed these detrimental effects, as evidenced by increased expression of PINK1, Parkin, PGC-1α, OPA1, DRP1, NDUFA10, UQCRSF1, and ATP5A in the cerebellum of YG8R mice compared to untreated YG8R controls. Moreover, ARC-18 treatment enhanced the phosphorylation of AMPKα and upregulated the expression of AMPKα, Beclin-1, and LC3B-II in the cerebellum of YG8R mice (Figure 1g and h, Figure S6A). These findings suggest that ARC-18 restored mitochondrial function and autophagy processes in FXN-deficient conditions.

ARC-18 Ameliorated Inflammation and its associated marker expression in FXN-deficient mice

To further elucidate the neuroprotective mechanisms of ARC-18, we evaluated

inflammation and related signaling molecules in both the serum and cerebellum of the experimental mice. The knockdown of FXN led to elevated serum malondialdehyde (MDA) levels and reduced concentrations of High-Density Lipoprotein Cholesterol (HDL-C), Creatine Kinase (CK), Aspartate Aminotransferase (AST), Alanine Aminotransferase (ALT), Albumin to Globulin Ratio (ALB/GLO ratio), and Urea in YG8R mice. Conversely, ARC-18 treatment reduced serum MDA and TG levels while increasing CK and AST/ALT ratios. In the cerebellum of YG8R mice, ARC-18 treatment enhanced Malondialdehyde (MDA), Hydrogen peroxide (H_2O_2), and Nitric oxide (NO) levels while decreasing GSH and IL-10 levels. Notably, ARC-18-treated YG8R mice exhibited increased expression of NeuN, HO-1, and GPX4 in cerebellar tissues compared to untreated YG8R mice (Figure S7A and B).

Proteomic Analysis Reveals ARC-18 Restores Mitochondrial Function and Alleviates FXN Deficiency-Associated Proteomic Dysregulation

Next, we performed mass spectrometry (MS) and proteomic analysis using shRNA FXN-KD N2a cells and cerebellum tissues from YG8R mice to further elucidate the mechanism of ARC-18. Proteomic profiling identified 5,864 proteins in the cerebellum, of which 1,895 were differentially expressed, including 847 upregulated and 1,048 downregulated proteins. Bioinformatics analysis using KEGG revealed that the downregulated proteins were significantly enriched in mitochondrial organization and translation pathways, suggesting that mitochondrial biosynthesis may be involved in the pathophysiological progression of FXN deficiency (Figure S4).

To further investigate the molecular mechanisms by which ARC-18 mitigates movement disorders in FXN-deficient animals, the proteomic analysis identified 416 differentially expressed proteins across WT, YG8R, and YG8R+ARC-18 groups (Figure 7A and 7B). These proteins were classified into two clusters (Figures 7C and 8B). KEGG enrichment and hierarchical clustering revealed

that ARC-18 treatment downregulated proteins involved in glycolysis, apoptosis, and nitrosative stress responses (Figure S5A). Conversely, ARC-18 upregulated pathways associated with mitochondrial electron transport, morphogenesis, protein ubiquitination, and proteolysis (Figure S5B). Molecular complex detection (MCODE) and heat maps predicted protein-protein interactions and highlighted trends in the alterations of these pathways (Figures S5A and C). To further characterize mitochondrial changes induced by ARC-18, we identified differentially expressed proteins related to mitochondrial structure and function. ARC-18 treatment significantly enhanced the expression of mitochondrial membrane structure proteins, transporter proteins, and oxidative phosphorylation-related complexes. These results suggest that ARC-18 activates PGC1α, potentially restoring mitochondrial protein imbalances caused by FXN deficiency and facilitating the recovery of motor function in YG8R mice.

References

1. Medras ZJH, Mostafa YM, Ahmed AAM, El-Sayed NM (2023) Arctigenin improves neuropathy via ameliorating apoptosis and modulating autophagy in streptozotocin-induced diabetic mice. CNS Neurosci Ther 29 (10):3068-3080. doi:10.1111/cns.14249
2. Zheng C, Li W, Ali T, Peng Z, Liu J, Pan Z, Feng J, Li S (2023) Ibrutinib Delays ALS Installation and Increases Survival of SOD1G93A Mice by Modulating PI3K/mTOR/Akt Signaling. Journal of Neuroimmune Pharmacology 18 (3):383-396. doi:10.1007/s11481-023-10068-9
3. McMackin MZ, Henderson CK, Cortopassi GA (2017) Neurobehavioral deficits in the KIKO mouse model of Friedreich's ataxia. Behav Brain Res 316:183-188. doi:10.1016/j.bbr.2016.08.053
4. He K, Nie L, Ali T, Liu Z, Li W, Gao R, Zhang Z, Liu J, Dai Z, Xie Y, Zhang Z, Liu G, Dong M, Yu Z-J, Li S, Yang X (2023) Adiponectin deficiency accelerates brain aging via mitochondria-associated neuroinflammation. Immunity & Ageing 20 (1):15. doi:10.1186/s12979-023-00339-7
5. Kádár A, Wittmann G, Liposits Z, Fekete C (2009) Improved method for combination of immunocytochemistry and Nissl staining. J Neurosci Methods 184 (1):115-118. doi:10.1016/j.jneumeth.2009.07.010
6. Zhang N, Wen Q, Ren L, Liang W, Xia Y, Zhang X, Zhao D, Sun D, Hu Y, Hao H, Yan Y, Zhang G, Yang J, Kang T (2013) Neuroprotective effect of arctigenin via upregulation of P-CREB in mouse primary neurons and human SH-SY5Y neuroblastoma cells. Int J Mol Sci 14 (9):18657-18669. doi:10.3390/ijms140918657

7. Li K, Gao L, Zhou S, Ma YR, Xiao X, Jiang Q, Kang ZH, Liu ML, Liu TX (2023) Erythropoietin promotes energy metabolism to improve LPS-induced injury in HK-2 cells via SIRT1/PGC1-α pathway. Mol Cell Biochem 478 (3):651-663. doi:10.1007/s11010-022-04540-y

8. Gong Q, Ali T, Hu Y, Gao R, Mou S, Luo Y, Yang C, Li A, Li T, Hao LL, He L, Yu X, Li S (2024) RIPK1 inhibition mitigates neuroinflammation and rescues depressive-like behaviors in a mouse model of LPS-induced depression. Cell Communication and Signaling 22 (1):427. doi:10.1186/s12964-024-01796-3

9. Gong Q, Li W, Ali T, Hu Y, Mou S, Liu Z, Zheng C, Gao R, Li A, Li T, Li N, Yu Z, Li S (2023) eIF4E phosphorylation mediated LPS induced depressive-like behaviors via ameliorated neuroinflammation and dendritic loss. Transl Psychiatry 13 (1):352. doi:10.1038/s41398-023-02646-5
