# Supplementary material for: Arctigenin derivative (ARC-18) improved mitochondrial dysfunction and ameliorated frataxin deficiency symptoms via PGC-1α signaling
Source: Genes Dis. 2025 Sep 1;13(4):101838. doi: 10.1016/j.gendis.2025.101838 (PMC13011025; doi:10.1016/j.gendis.2025.101838)
Supplement: Multimedia component 5 [file mmc5.pdf]

1. Procedures for the synthesis of ARC-18

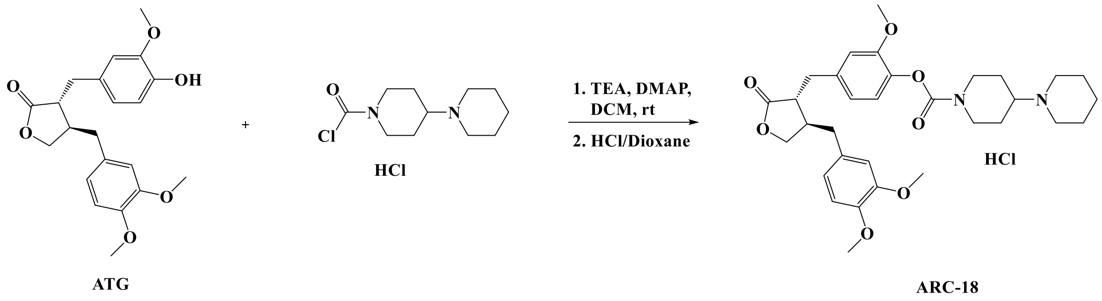

A solution of compound **ATG** (Arctigenin, 372.4 mg, 1 mmol) and 1-chloroformyl-4-piperidine hydrochloride (320.6 mg, 1.2 mmol) in anhydrous dichloromethane (20 mL) was added 4-dimethylaminopyridine (12.2 mg, 0.1 mmol) and triethylamine (0.1 mL, 1.6 mmol) in order, and the mixture was stirred at the room temperature for 12 h. Then the mixture was washed by dilute hydrochloric acid and saturated sodium bicarbonate solution in turn, dried over Na_2SO_4, filtered, concentrated under vacuum and purified by silica gel chromatography. The obtained was directly dissolved in anhydrous dioxane and added dioxane solution with hydrogen chloride (10 mL, 2 mol/L) and stirred at the room temperature for 2 h. Then, concentrated under vacuum to give **ARC-18** (440.23 mg, 73%).

2. Identification results for ARC-18

(1) Mass spectrometry

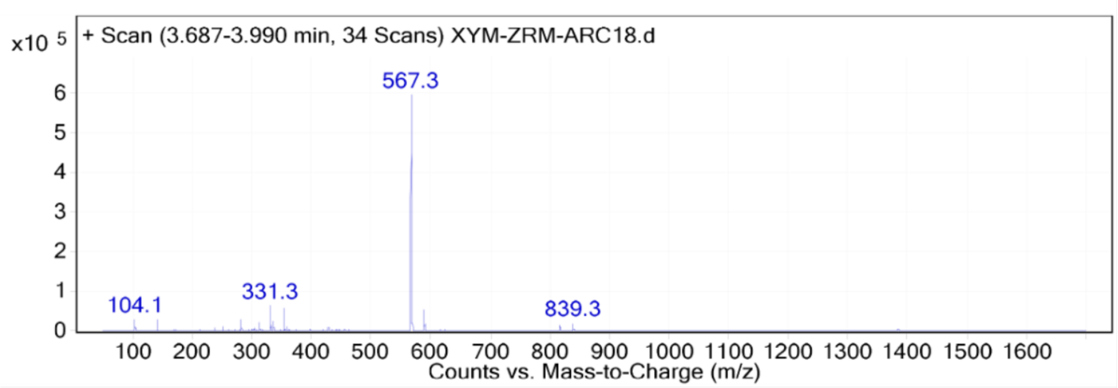

(2) ^{13}C Nuclear Magnetic Resonance Spectra

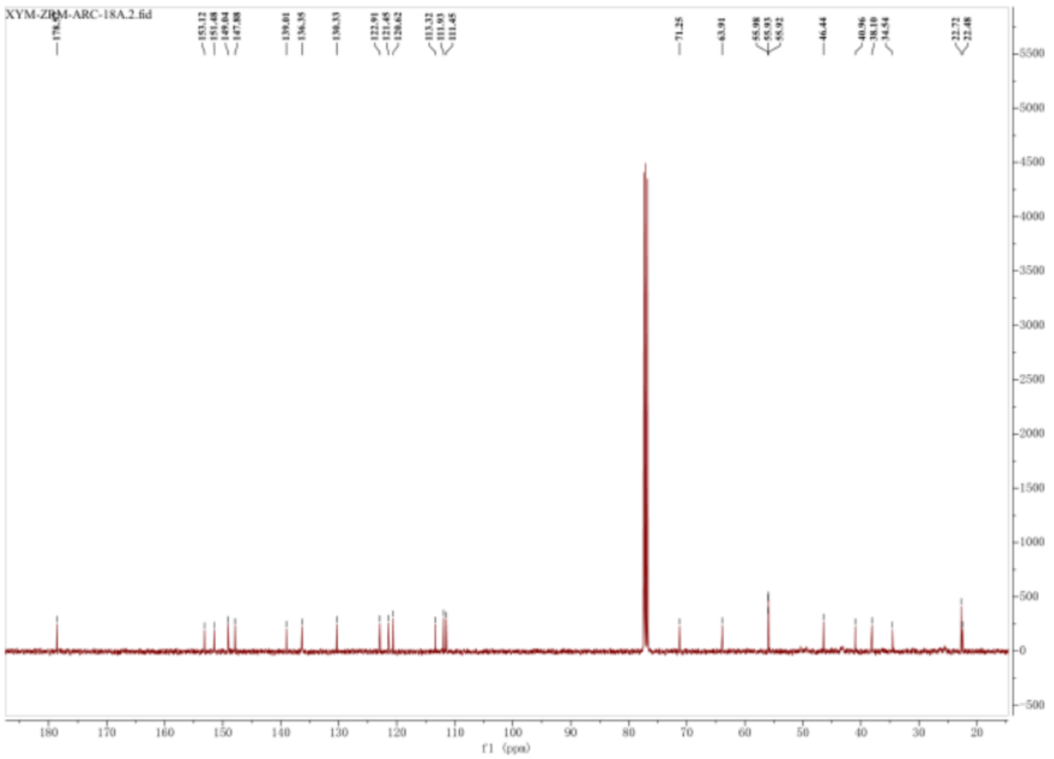

^{13}C NMR (101 MHz, CDCl3) δ 178.57, 153.12, 151.48, 149.04, 147.88, 139.01, 136.57, 130.33, 122.91, 121.45, 120.62, 113.32, 111.92, 111.46, 71.25, 63.91, 55.98, 55.93, 55.93, 55.92, 55.92, 46.44, 46.44, 40.96, 40.96, 38.10, 34.54, 22.72, 22.72, 22.71, 22.71, 22.47.

(3) ^{1}H Nuclear Magnetic Resonance Spectra

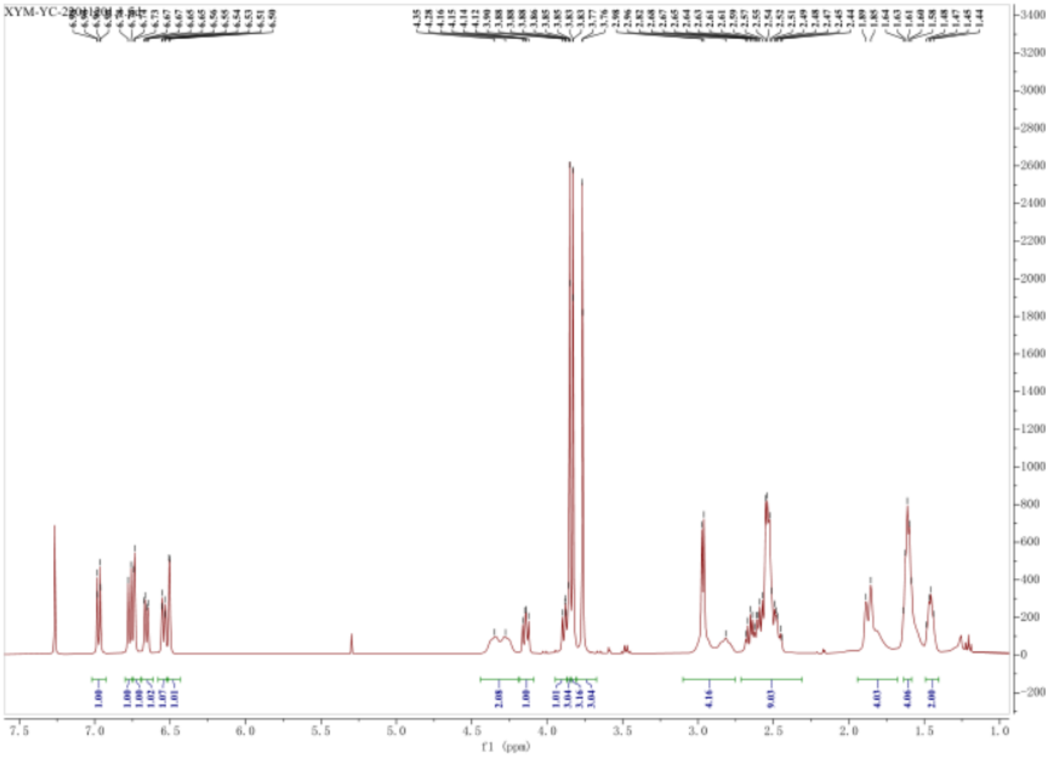

^{1}H NMR (400 MHz, CDCl3) δ 6.97 (dd, J = 8.0, 1H), 6.77 (d, J = 8.1 Hz, 1H), 6.74 (d, J = 1.9 Hz,

1H), 6.66 (dd, *J* = 8.0, 1.9 Hz, 1H), 6.54 (dd, *J* = 8.1, 2.0 Hz, 1H), 6.51 (d, *J* = 2.0 Hz, 1H), 4.41– 4.21 (m, 2H), 4.14 (dd, *J* = 9.0, 6.9 Hz, 1H), 3.91 – 3.87 (m, 1H), 3.85 (s, 3H), 3.83 (s, 3H), 3.77 (s, 3H), 3.01– 2.75(m, 4H), 2.71 – 2.40 (m, 9H), 1.92 – 1.74 (m, 4H), 1.66– 1.52 (m, 4H), 1.49 – 1.39 (m, 2H).
